# Ethical issues related to cohorts and bio-banks research

**DOI:** 10.1186/1753-6561-7-S5-O21

**Published:** 2013-08-30

**Authors:** Nandini K Kumar

**Affiliations:** 1 National Institute of Epidemiology, Chennai, India

## 

The codes and practices of professional morality had been enunciated centuries ago in the traditional Indian systems of medicine. The modern code of medical and research ethics has its origin in the Nuremberg Code, Universal Declaration of Human Rights, Code of Medical Ethics by Medical Council of India, World Health Assembly Helsinki Declaration, Belmont report and the Revised Council for International Organizations of Medical Sciences (CIOMS) guidelines. The revised Indian Council of Medical Research (ICMR) guidelines have been adopted in 2006.

The ethical principles of autonomy, justice, non-maleficense and beneficense are applicable to any research undertaken. Careful attention has to be given to the processes of taking consent, assent from minors, re-consent, community engagement, risk-benefit assessment, interventions, data management and follow up. Special concerns for cohorts include the difficulty in conveying results due to the absence of immediate benefits and unpredictable susceptibility due to genetic variations. There are issues of confidentiality and stigmatization due to the allotment of unique identification. There is a need for translational research in cohort studies to understand the priorities, validity and applicability in different cultural settings and the role of community based participatory research. There are concerns in research methodology due to shifting from acute paradigm to chronic paradigm, involvement of special population groups.

Bio-banks have advantages such as advancement of scientific knowledge, present and future medical benefits for the individual, improved pharmaco-genomic understanding and commercial benefits through patents. The major ethical concerns revolve around collection, storage and management of samples. Human dignity, right to share benefits, intellectual property rights, access to personal data, sample sharing by researchers, movement of samples outside the country, samples from deceased or non-traceable persons are all issues which has to be carefully thought out. Breach of confidentiality and unwanted information flow can result in anxiety, depression, social stigma and discrimination. There are also issues of cultural and social acceptability for taking samples.

The ICMR has issued guidelines for DNA banking in 2006 for primary and secondary use of samples. A draft DNA Profiling Bill, 2007 is under the consideration of the Indian Parliament which looks to legalize the collection and analysis of DNA samples and to create a balance between the constitutional rights of an individual and the public interest along with accountability and transparency in the practice of DNA collection and testing. It will be essential to establish standards for laboratories, staff qualifications, training, proficiency testing, collection of body substances, custody trail from collection to reporting and a data Bank with policies of use and access to information therein, its retention and deletion. Thus genetic technology faces ethical challenges in the clinical, social, economic, scientific, legal and political domains.

